# Carotid artery intima-media thickness, HDL cholesterol levels, and gender associated with poor visual acuity in patients with branch retinal artery occlusion

**DOI:** 10.1371/journal.pone.0240977

**Published:** 2020-10-22

**Authors:** Masayuki Yasuda, Hajime Sato, Kazuki Hashimoto, Urara Osada, Takehiro Hariya, Hiroko Nakayama, Toshifumi Asano, Noriyuki Suzuki, Tatsu Okabe, Mai Yamazaki, Megumi Uematsu, Masanori Munakata, Toru Nakazawa

**Affiliations:** 1 Department of Ophthalmology, Tohoku University Graduate School of Medicine, Sendai, Miyagi, Japan; 2 Yaotome Sato Hajime Eye Clinic, Miyagi, Japan; 3 Department of Ophthalmology, JR Sendai Hospital, Sendai, Miyagi, Japan; 4 Department of Ophthalmology, Tohoku Rosai Hospital, Sendai, Miyagi, Japan; 5 Seiryo Eye Clinic, Miyagi, Japan; 6 Division of Hypertension & Research Center for Lifestyle-Related Disease, Tohoku Rosai Hospital, Sendai, Miyagi, Japan; 7 Department of Ophthalmic Imaging and Information Analytics, Tohoku University Graduate School of Medicine, Sendai, Miyagi, Japan; 8 Department of Retinal Disease Control, Tohoku University Graduate School of Medicine, Sendai, Miyagi, Japan; 9 Department of Advanced Ophthalmic Medicine, Tohoku University Graduate School of Medicine, Sendai, Miyagi, Japan; VU University Medical Center, NETHERLANDS

## Abstract

**Purpose:**

To investigate factors associated with poor visual acuity (VA) in branch retinal artery occlusion (BRAO).

**Methods:**

This was a retrospective cross-sectional study of 72 eyes with BRAO of 72 patients. For statistical comparison, we divided the patients into worse-VA (decimal VA < 0.5) and better-VA (decimal VA > = 0.5) groups. We examined the association of clinical findings, including blood biochemical test data and carotid artery ultrasound parameters, with poor VA.

**Results:**

Median age, hematocrit, hemoglobin and high-density lipoprotein (HDL) differed significantly between the groups (*P* = 0.018, *P* < 0.01, *P* < 0.01, and *P* = 0.025). There was a tendency towards higher median IMT-Bmax in the worse-VA group (worse-VA vs. better-VA: 2.70 mm vs. 1.60 mm, *P* = 0.152). Spearman’s rank correlation test revealed that logMAR VA was significantly correlated to IMT-Bmax (rs = 0.31, *P* < 0.01) and IMT-Cmax (rs = 0.24, *P* = 0.035). Furthermore, logMAR VA was significantly correlated to HDL level (rs = -0.33, *P* < 0.01). Multivariate logistic regression analysis revealed that IMT-Bmax (odds ratio [OR] = 2.70, *P* = 0.049), HDL level (OR = 0.91, *P* = 0.032), and female gender (OR = 15.63, *P* = 0.032) were independently associated with worse VA in BRAO.

**Conclusions:**

We found that increased IMT-Bmax, decreased HDL, and female sex were associated with poor VA in BRAO patients. Our findings might suggest novel risk factors for visual dysfunction in BRAO and may provide new insights into the pathomechanisms underlying BRAO.

## Introduction

Branch retinal artery occlusion (BRAO), one of the most common retinal vascular disorders, can cause sudden vision loss and visual field defects [1]. Although various treatments have been proposed [2–4], there are not yet any proven, effective treatments for BRAO once it occurs. The visual prognosis for BRAO is generally better than for central retinal artery occlusion (CRAO) [1], but nevertheless, 40 to 50% of BRAO cases show decimal visual acuity (VA) worse than 0.5 [5, 6]. Moreover, it has been reported that when VA at presentation is 0.4 or worse, only 25% of BRAO cases improve to a VA of 0.5 or better [5]. Thus, the visual prognosis for BRAO tends to depend on the initial VA. Factors associated with the poor prognosis in these cases remain to be determined, although systemic conditions associated with atherosclerosis are likely candidates, being well-known risk factors for the development of BRAO. There is thus a need to identify clinical indicators associated with a poor visual prognosis in BRAO, in order to establish preventive therapy for BRAO and associated decreased visual function.

Like other retinal occlusive disorders, such as CRAO and retinal vein occlusion (RVO), metabolic disorders such as hypertension and dyslipidemia [5], as well as lifestyle factors such as smoking [7], are involved in the development of BRAO. In eyes with BRAO, hypoperfusion of the retina, typically caused by emboli, causes retinal ischemic damage resulting in irreversible visual dysfunction. The most common source of emboli in BRAO is plaque in the carotid artery. Hayreh et al. examined the prevalence of carotid artery disease in 127 eyes with BRAO of 119 patients and found that 31% of BRAO patients had > 50% stenosis in the carotid artery and had plaque in the carotid artery on the affected side [8]. Therefore, screening for carotid artery disease is important to prevent the development of BRAO as well as other ocular occlusive disorders.

Carotid artery disease can be assessed with ultrasonography (US) [9]. Carotid US can evaluate atherosclerotic changes quantitatively by measuring intima-media thickness (IMT) [10]. Studies have shown that IMT is a good predictor for the risk of stroke [11–14] and cardiovascular diseases [15–17]. In addition, IMT in the common carotid artery has been demonstrated to be correlated to cerebral blood flow [18]. Furthermore, carotid artery disease has been reported to contribute to various ocular arterial occlusive disorders [8]. However, the relationship between carotid artery disease and visual dysfunction in BRAO remains unclear. In this study, we aimed to identify clinical factors, including carotid IMT, associated with decreased VA in BRAO.

## Patients and methods

This was a retrospective, cross-sectional, observational case series. We reviewed medical records from January 2011 to December 2016. All subjects had non-arteritic BRAO and were enrolled at Tohoku Rosai Hospital. BRAO was diagnosed based on fundus findings (i.e. retinal whitening due to ischemic changes) and fluorescence angiography findings (delayed arterial filling and a corresponding non-perfusion area). We only included eyes with BRAO which affected a part of the macular area. The exclusion criteria were arteritic BRAO, the presence of systemic inflammatory diseases such as systemic lupus erythematosus, and retinal diseases other than BRAO. The institutional review board of the Tohoku Rosai Hospital approved this study (approval number: #18–31). The research was conducted according to the provisions of the Declaration of Helsinki, 1995 (as revised in Edinburgh, 2000).

The subjects underwent a baseline ophthalmic examination, including measurement of visual acuity and intraocular pressure (IOP) and a slit lamp examination. Blood pressure (BP) was measured in each patient. Blood samples were collected from each patient, and complete blood count, hemoglobin A1c (HbA1c), lipids (i.e., triglycerides, high-density lipoprotein [HDL], and low-density lipoprotein [LDL]) were measured with automated standardized laboratory techniques. To evaluate renal function, the estimated glomerular filtration rate (eGFR) was calculated based on the serum creatinine level using the following formula: 194 × Cr −1.094 × age −0.287 (−0.739 if female [Cr: serum creatinine]) [19].

### Carotid artery ultrasound parameters

IMT, which is commonly used as an indicator of systemic atherosclerotic changes, was measured bilaterally in ultrasound B-scans of the carotid arteries with the Nemio 30 device (Toshiba) as previously described [10, 20, 21]. The carotid arteries were scanned in transverse sections at the origin of the common carotid arteries, the carotid sinuses, and the internal and external carotid arteries. Then, the ultrasound probe was rotated to obtain longitudinal images of the carotid walls. The ultrasound B-scans visualized the double-line pattern on both the near and the far wall of the carotid artery. The double-line pattern was defined as the intima-media complex. We then measured intima-media complex thickness (IMT). We measured IMT in the common carotid artery and carotid bifurcation and recorded the highest value for IMT in the near and far wall as IMT-Cmax in the common carotid artery and IMT-Bmax in the carotid bifurcation (S1 and S2 Figs).

### Statistical analyses

To identify associations between VA and clinical features, the eyes in this study were classified into two groups based on VA: the better VA group, with a VA of 0.5 or better, and the worse VA group, with a VA of worse than 0.5. The cut-off value of 0.5 was based on previous reports [5, 6]. Decimal VA was converted to the logMAR scale for statistical analysis. Continuous variables were expressed as the median (interquartile range [IQR]). The Mann-Whitney U test and Fisher’s exact test were used to evaluate differences in variables between the groups. The Wilcoxon-signed rank test was used to compare IMT-Bmax and IMT-Cmax between affected and unaffected sides of BRAO. Spearman’s rank correlation test was used to estimate relationships between logMAR VA and the variables. A multivariate logistic regression analysis was used to investigate factors associated with worse VA in BRAO. The pROC package in R software (version 1.15.3) was used to perform a receiver operating characteristic (ROC) curve analysis to assess the ability to discriminate worse VA in BRAO. All statistical analyses were performed with R software (version 3.6.1) [22]. Differences were considered significant at *P* < 0.05.

## Results

Table 1 shows the demographics of the subjects. This study enrolled 72 eyes with BRAO of 72 patients (56 male: 16 female, median age: 68.5 years ([IQR] 57.8–75.3). Median age, hematocrit (Hct) and hemoglobin (Hgb) differed significantly between the male and female subjects (*P* = 0.001 and *P* < 0.001, respectively). Systemic BP (SBP), diastolic BP (SBP), HbA1c, and lipid levels, which are involved in the development of atherosclerotic changes, did not differ between the male and female subjects. Both the median IMT-Bmax and IMT-Cmax were significantly higher in the male subjects than the female subjects (*P* < 0.001 and *P* < 0.022, respectively). In addition, we compared IMT-Bmax and IMT-Cmax between the affected and unaffected sides of BRAO within the same subjects (S1 Table). The analyses showed that IMT-Cmax was significantly higher on the BRAO side, while IMT-Bmax was not significantly different.

**Table 1:** Demographics and clinical characteristics of the BRAO patients.

	All (N = 72)	Male (N = 56)	Female (N = 16)	*P* value
Age (years)	68.5 [58.0, 76.0]	69.0 [59.0, 75.0]	66.0 [47.8, 78.0]	0.823
Laterality of BRAO = R (%)	45 (62.5)	35 (62.5)	10 (62.5)	1.000
VA (logMAR)	0.05 [-0.00, 0.24]	0.90 [0.70, 1.00]	0.60 [0.34, 1.05]	0.305
Days from onset	1.00 [0.75, 3.00]	1.00 [0.75, 3.00]	1.50 [0.75, 3.50]	0.451
Smoking history (%)	24 (33.3)	22 (39.3)	2 (12.5)	0.070
History of hypertension (%)	50 (69.4)	38 (67.9)	12 (75.0)	0.761
SBP (mmHg)	144.0 [127.0, 156.3]	144.0 [130.5, 153.8]	142.0 [123.0, 174.5]	0.670
DBP (mmHg)	82.5 [70.8, 93.0]	82.0 [69.8, 92.3]	85.5 [71.8, 111.8]	0.378
HbA1c (%)	5.75 [5.50, 6.10]	5.80 [5.50, 6.10]	5.65 [5.45, 6.00]	0.41
Hct (%)	41.4 [38.8, 44.1]	42.5 [40.0, 44.4]	38.6 [37.6, 40.9]	0.001**
Hgb (g/dl)	14.1 [13.1, 15.0]	14.6 [13.7, 15.3]	12.9 [12.5, 13.7]	<0.001***
HDL (mg/dl)	48.0 [38.8, 54.0]	47.0 [37.0, 53.3]	50.0 [42.5, 56.3]	0.180
LDL (mg/dl)	103.0 [81.8, 127.3]	102.5 [81.0, 125.5]	112.0 [92.8, 141.3]	0.165
TG (mg/dl)	117.0 [87.3, 158.8]	113.5 [88.8, 158.8]	125.0 [75.5, 155.3]	0.968
eGFR (mL/min/1.73m2)	67.6 [56.7, 76.5]	67.6 [55.8, 76.5]	66.6 [57.0, 75.1]	0.924
CRP (mg/dl)	0.13 [0.05, 0.29]	0.11 [0.05, 0.33]	0.15 [0.05, 0.19]	0.650
IMT-Bmax (mm)	1.65 [1.10, 2.52]	1.95 [1.37, 2.70]	1.05 [0.90, 1.50]	<0.001***
IMT-Cmax (mm)	1.20 [0.90, 2.00]	1.25 [0.97, 2.60]	1.00 [0.88, 1.20]	0.022*

Continuous variables are expressed as median [IQR]. The Man-Whitney U test was used to compare continuous variables between groups. Fisher’s exact test was used to compare categorical variables between groups.

*P < 0.05,

**P < 0.01,

***P < 0.001.

VA: visual acuity, SBP: systolic blood pressure, DBP: diastolic blood pressure, HbA1c: glycated hemoglobin, Hct: hematocrit, Hgb: hemoglobin, HDL: high-density lipoprotein, LDL: low-density lipoprotein, TG: triglyceride, eGFR: estimated glomerular filtration rate, CRP: C-reactive protein.

Table 2 shows differences in values between the better- and worse-VA groups. Fifty-eight patients had VA better than 0.5 (better-VA group), and 14 patients had VA less than 0.5 (worse-VA group). Median age, hematocrit, hemoglobin, and HDL differed significantly between the groups (*P* = 0.018, *P* < 0.01, *P* < 0.01 and *P* = 0.025, respectively). Systemic blood pressure, diastolic blood pressure, and HbA1c, which are involved in the development of atherosclerotic changes, did not differ between the groups. In addition, days from onset did not differ between the groups (*P* = 0.730). Although there was no statistically significant difference in IMT-Bmax, there was a tendency towards higher IMT-Bmax in the worse-VA group (worse-VA vs. better-VA: 2.70 [1.35, 2.88] vs. 1.60 [1.10, 2.38], *P* = 0.152).

**Table 2:** Differences in values between the VA groups.

	VA ≥ 0.5 (N = 58)	VA < 0.5 (N = 14)	*P* value
Age (years)	67.0 [57.3, 72.8]	77.0 [68.5, 82.8]	0.018*
Gender = F/M (%)	11/47 (19.0/81.0)	5/9 (35.7/64.3)	0.280
Laterality of BRAO = R (%)	35 (60.3)	10 (71.4)	0.645
VA (logMAR)	0.00 [-0.00, 0.15]	1.00 [0.61, 1.28]	<0.001***
Days from onset	1.00 [0.25, 3.00]	1.00 [1.00, 2.50]	0.730
Smoking history (%)	21 (36.2)	3 (21.4)	0.359
History of hypertension	41 (70.7)	9 (64.3)	0.886
SBP (mmHg)	145.0 [128.3, 156.8]	133.5 [115.0, 149.5]	0.147
DBP (mmHg)	83.5 [71.0, 94.5]	81.0 [67.0, 89.8]	0.503
HbA1c (%)	5.80 [5.50, 6.10]	5.60 [5.25, 5.85]	0.199
Hct (%)	42.2 [40.2, 44.2]	38.8 [36.9, 40.0]	<0.01**
Hgb (g/dl)	14.5 [13.6, 15.0]	13.1 [12.2, 13.6]	<0.01**
HDL (mg/dl)	49.0 [42.0, 54.8]	39.0 [30.3, 50.0]	0.025*
LDL (mg/dl)	114.5 [88.3, 130.8]	87.5 [81.0, 106.5]	0.159
TG (mg/dl)	121.0 [96.5, 177.0]	91.5 [76.5, 134.5]	0.096
eGFR (mL/min/1.73m2)	67.6 [56.9, 75.9]	69.2 [51.4, 77.3]	0.977
CRP (mg/dl)	0.11 [0.05, 0.23]	0.20 [0.09, 0.47]	0.084
IMT-Bmax (mm)	1.60 [1.10, 2.38]	2.70 [1.35, 2.88]	0.152
IMT-Cmax (mm)	1.20 [0.90, 2.08]	1.20 [1.00, 1.48]	0.853

Continuous variables are expressed as median [IQR]. The Man-Whitney U test was used to compare continuous variables between groups. Fisher’s exact test was used to compare categorical variables between groups.

*P < 0.05,

**P < 0.01,

***P < 0.001.

VA: visual acuity, SBP: systolic blood pressure, DBP: diastolic blood pressure, HbA1c: glycated hemoglobin, Hct: hematocrit, Hgb: hemoglobin, HDL: high-density lipoprotein, LDL: low-density lipoprotein, TG: triglyceride, eGFR: estimated glomerular filtration rate, CRP: C-reactive protein.

**Fig 1:**
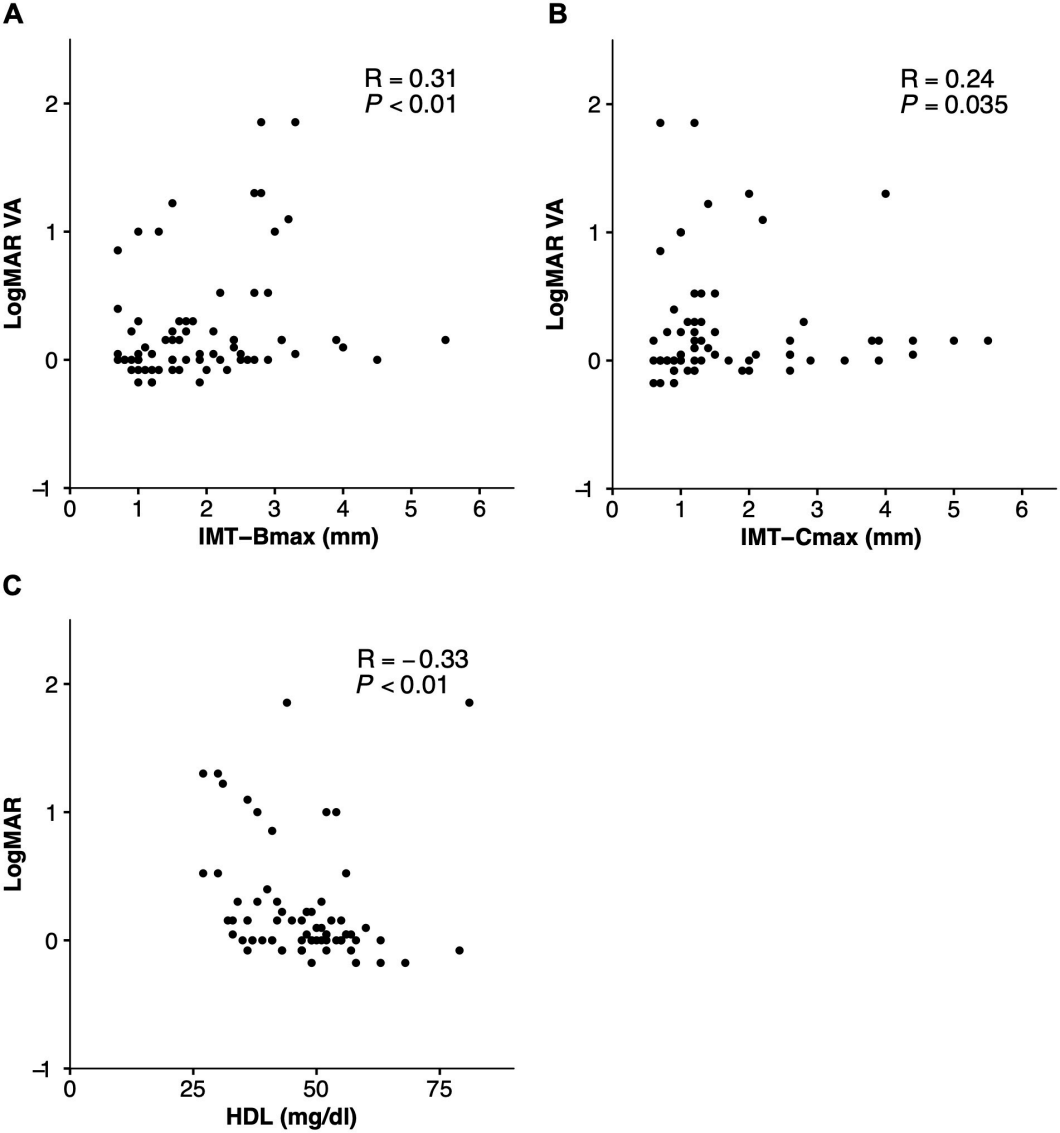
Correlation of visual acuity with carotid ultrasound parameters and HDL level in BRAO. Scatterplot showing correlations of logMAR VA in BRAO at initial presentation with carotid ultrasound parameters (**A**: IMT-Bmax, **B**: IMT-Cmax) and HDL level (**C**). Spearman’s rank correlation test was performed to assess correlation coefficients (rs) and statistical significance.

**Fig 2:**
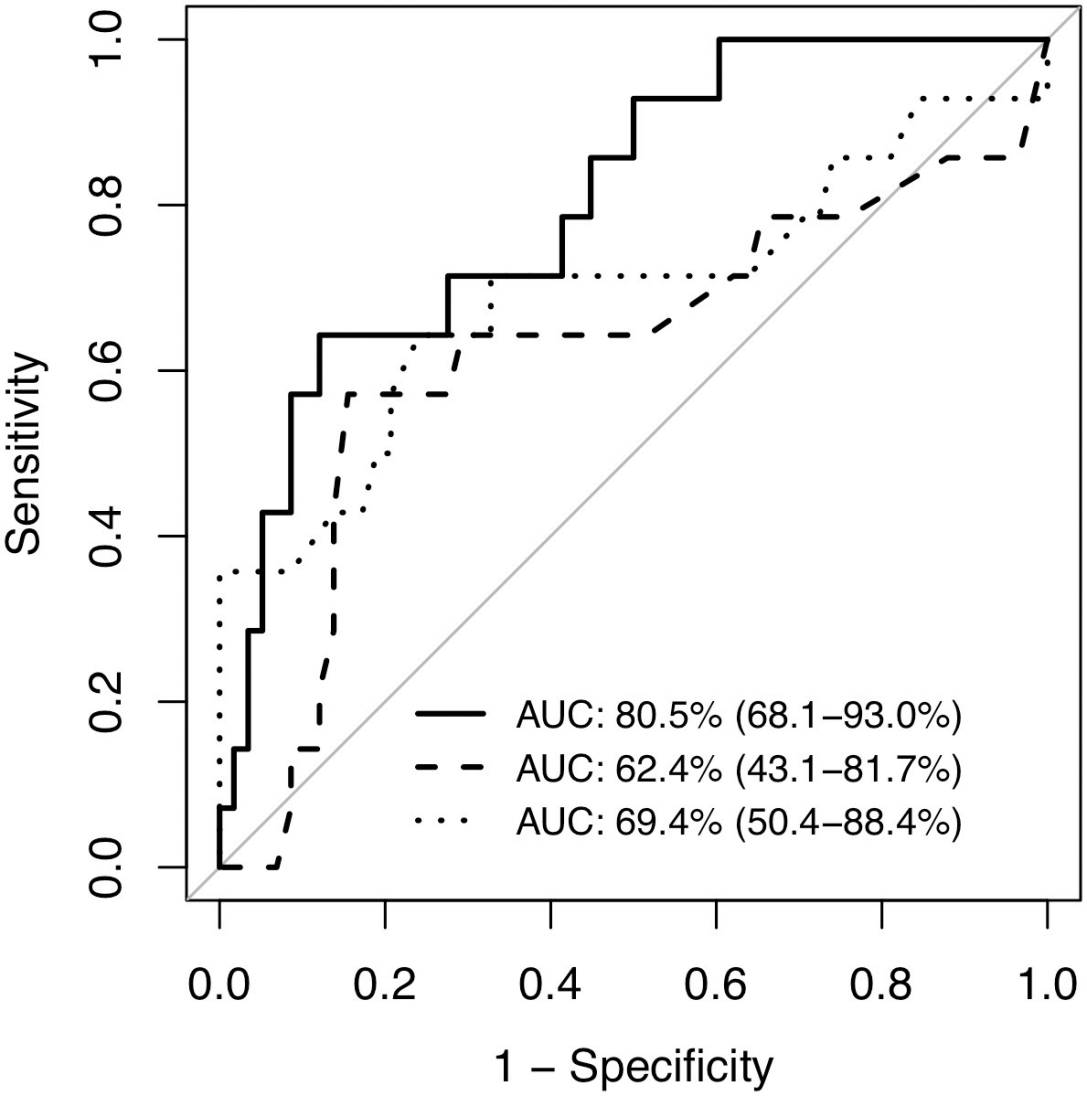
Discriminative power of age, gender, HDL, and IMT-Bmax for poor visual acuity in BRAO. ROC analysis of HDL (dotted line) and IMT-Bmax (dashed line). The model included age, gender, HDL, and IMT-Bmax (solid line). The area under the curve (AUC) is plotted.

Spearman’s rank correlation test revealed that logMAR VA was significantly correlated to IMT-Bmax (rs = 0.31, *P* < 0.01) and IMT-Cmax (rs = 0.24, *P* = 0.035) (Fig 1A and 1B). Furthermore, logMAR VA was significantly correlated to HDL level (rs = -0.33, *P* < 0.01) (Fig 1C). Multivariate logistic regression analysis revealed that IMT-Bmax (odds ratio [OR] = 2.76, *P* = 0.042), HDL level (OR = 0.91, *P* = 0.032), and female gender (OR = 18.92, *P* = 0.013) were independently associated with worse VA in BRAO (Table 3). Receiver operating characteristic (ROC) analysis indicated that the area under the curve (AUC) for IMT-Bmax and HDL was 62.4% (95% confidence interval [CI]: 43.1–81.7%) and 69.4% (95% CI: 50.4–88.4%), respectively. Further ROC analysis demonstrated that a model including age, gender, IMT-Bmax, and HDL had a high discriminative power (80.5% [95% CI: 68.1–93.0%]) for worse VA in BRAO (Fig 2).

**Table 3:** Factors independently associated with poor visual acuity at initial presentation in BRAO.

Variable		Adjusted OR (95% Cl)	*P* value
Response	Explanatory		
Poor VA (decimal VA < 0.5)	Age	1.03 (0.97–1.10)	0.309
	Gender (female)	18.92 (1.86–192.29)	0.013*
	HbA1c	0.35 (0.08–1.57)	0.172
	CRP	5.27 (0.83–33.41)	0.078
	HDL	0.91 (0.83,0.99)	0.032*
	IMT-Bmax	2.76 (1.04,7.37)	0.042*

Multivariate logistic regression analysis. OR: Odds ratio. Nagelkerke’s R-squared = 0.42

*P < 0.05.

HbA1c: glycated hemoglobin, CRP: C-reactive protein, HDL: high-density lipoprotein.

## Discussion

BRAO is a common ocular artery disorder, and there is no proven effective treatment [23]. Although systemic conditions involved in the development of atherosclerosis are known to also play a crucial role in the pathogenesis of BRAO, the risk factors for poor VA have not been determined. In the current study, we investigated the relationships between VA and systemic parameters, including carotid US, and found that IMT-Bmax, as well as low HDL, were associated with poor VA in BRAO at the initial presentation.

Studies have examined changes in VA among patients with BRAO and demonstrated that VA at the initial presentation was significantly correlated to visual prognosis after BRAO [5, 6]. Generally, the prognosis for VA in BRAO is far better than it is for central retinal artery occlusion, but around 40% of BRAO patients have been reported to present with VA worse than 0.5 [5]. In the current study, 21% of patients presented with VA worse than 0.5, a rate relatively lower than in previous reports. VA in BRAO tends to improve naturally with time, thus, this difference may be partly due to the inclusion of subjects who were examined later after onset than in previous studies. Mason et al. investigated 52 eyes of 52 patients with BRAO and demonstrated that only 25% of those that presented with VA of 0.4 or worse eventually improved to 0.5 or better [5]. Yuzurihara et al. investigated VA in 30 eyes with BRAO at the initial and follow-up visits in a Japanese population. This study also showed a significant correlation between initial and final VA in BRAO [6]. This suggests that poor VA at the time of presentation may be an important risk factor for worse VA outcomes in BRAO. Therefore, our study provides information that may be useful for the prevention of vision loss due to BRAO, and is the first to report various risk factors for poor VA at the time of presentation.

In this study, we found that BRAO patients with higher IMT-Bmax were more likely to have worse VA at the initial presentation. IMT measured with US represents the luminal-intimal and the medial-adventitial interfaces, corresponding well with IMT measured in histological specimens [24]. Increased carotid IMT is considered as a surrogate marker for general atherosclerosis [25]. In addition, increased IMT is reportedly associated with the presence of carotid plaques [26]. Many studies have shown that increased carotid IMT is a predictive factor for cerebro-cardiovascular events [27, 28]. A systematic review and meta-analysis demonstrated that the relative risk for myocardial infarction and stroke was 1.26 (95% CI, 1.21 to 1.30) and 1.32 (95% CI, 1.27 to 1.38) per 1–standard deviation difference in common carotid artery IMT [29]. There have been no studies on the relationship between carotid IMT and BRAO, and we were unable to investigate the association of IMT with the development of BRAO due to the lack of controls without BRAO. However, carotid artery disease has been reported to play an important role in the development of BRAO. The most common cause of BRAO is emboli formed from plaque in the carotid artery [7]; 64% of BRAO cases show the presence of plaque in the affected retinal artery [7].

In the current study, IMT-Bmax showed a significant association with worse VA in BRAO, while IMT-Cmax did not. The carotid bifurcation is prone to early development of atherosclerotic changes. Blood flow turbulence occurring in the carotid bifurcation is one of the main sources of atherosclerotic plaque, which can become embolic debris and thrombi leading to cerebral infarction [30], suggesting that IMT-Bmax is more closely associated with the pathogenesis of BRAO. In the current study, median IMT-Bmax was 2.70 mm in the subjects overall. This value was higher than that in patients without coronary artery stenosis (CAS) and similar to the value in Japanese patients with CAS and at least 3 diseased vessels [20]. In a previous investigation of the relationship between carotid atherosclerotic changes and optic nerve head (ONH) circulation, blowout time (BOT), a pulse-waveform parameter representing blood supply in the ONH, measured with laser speckle flowgraphy (LSFG), was independently associated with abnormal carotid IMT thickening [31]. Furthermore, we previously found that IMT-Cmax was correlated to decreased ONH blood flow and the presence of retinopathy in type 2 diabetes patients [32]. These results suggest that changes in the carotid artery contribute to changes in ocular microcirculation in BRAO. Thus, hypoperfusion in the retina makes it more vulnerable to decreased ocular microcirculation, leading to poor VA in BRAO patients with high IMT in the carotid bifurcation.

Another interesting finding of this study was the association between decreased HDL and worse VA in BRAO. It is well established that low HDL levels are associated with increased risk for cardiovascular diseases [33, 34]. Several studies have demonstrated that lower HDL levels increase the risk for stroke [35, 36]. Furthermore, Munakata et al. revealed that low HDL was associated with stroke, as well as cardiovascular events, in a Japanese population-based cohort study [37, 38]. HDL has been shown to elicit multiple anti-atherogenic effects by acting as an anti-oxidant and anti-inflammatory [39–44]. This suggests that decreased HDL is associated with plaque formation in the carotid artery and retinal endothelial damage, which are implicated in the development of BRAO and vulnerability of the retina to ischemic insult. Although the underlying mechanism leading to reduced HDL remains unclear, multiple factors, including genetics [45], poor glycemic control [46], and smoking [47, 48] have been reported to be involved. Although reduced HDL might be ameliorated by changes to modifiable risk factors such as smoking and diabetes, there are no currently available drugs for the treatment of reduced HDL. The development of such drugs might lead to more effective methods of preventing poor VA outcomes in BRAO.

In addition to IMT-Bmax and HDL, we found that female patients were more likely to have worse VA in BRAO. Several studies have shown gender differences in stroke [49–51]. A systematic review that included mainly Western European surveys reported that early mortality after stroke was higher in women than in men [52]. In addition, women are more likely to be disabled after stroke than men [53, 54]. In Japan, the Hisayama study, a population-based prospective cohort study, demonstrated that the age-standardized stroke mortality rate was higher in men [55]. On the other hand, the early mortality rate and functional outcomes after acute stroke have shown to be poorer in women in Japan. The Oyabe study conducted in Japan showed that the 28-day case fatality rates for stroke were significantly higher among the female patients younger than 65 years in 1987–1991 [56]. Furthermore, a recent nationwide multicenter stroke registration study in Japan (the Japan Standard Stroke Registry Study: JSSRS) have shown that acute care hospital stays were longer and stroke outcomes at discharge were worse in women than in men [57]. Our study finding that female BRAO patients were more likely to have poor VA is similar to these trends in the early mortality rate and functional outcomes after stroke in Japan.

Studies suggest that sex steroid hormones, especially estrogen, play important roles in sex differences in stroke [58]. Estrogen has anti-oxidative, anti-inflammatory, and anti-apoptotic effects [59–61], which are thought to contribute to the lower prevalence of stroke in women at younger ages. Estrogen receptors are present in the human retina [62], and various animal-model studies have shown the protective effect of estrogen against retinal injury, including ischemia/reperfusion injury [63–67]. Therefore, the retina of postmenopausal women might be more susceptible to ischemic damage caused by BRAO. In the current study, 2 of 5 female patients with worse VA were in their 40s and probably premenopausal. Factors besides estrogen, such as genetics, are also thought to be associated with sex differences in stroke [58]. Of the two female patients in their 40s, one had atrial fibrillation with low IMT-Cmax and IMT-Bmax (0.7 mm and 0.7 mm), and cardiac embolism was thus suspected as the cause of BRAO. Although the exact mechanism of cardioembolic stroke remains unknown, it is generally more severe than other types of ischemic stroke [68]. The other female patient in her 40s with poor VA had uncontrolled hyperlipidemia and hypertension. A previous study showed that women with metabolic syndrome were more likely to develop early subclinical atherosclerosis than men [69]. Moreover, metabolic syndrome doubled the risk of ischemic stroke in women while it had no effect in men [70], suggesting that women are more susceptible to metabolic syndrome.

Several studies have reported gender differences in retinal morphology and ocular microcirculation. For example, one study examined 80 eyes of 90 healthy patients (43 women, 47 men) using spectral-domain optical coherence tomography [71], and found that central and parafoveal retinal thickness were lower in women than men, although there were no differences in foveal morphology. Kobayashi et al. examined gender differences in ocular microcirculation with LSFG and demonstrated that BOT, the aforementioned pulse-waveform parameter, in the ONH tissue area (BOT-T) was significantly lower in women than in men among subjects aged over 41 years [72]. Furthermore, the difference in BOT-T between genders was greater among subjects aged over 61 years. Although we did not measure ocular LSFG in the current study, worse VA outcomes in female BRAO patients might be associated with gender differences in ocular microcirculation. We thus suggest that more attention should be paid to the needs of older female patients at risk of developing BRAO.

Our study had several limitations. First, it was cross-sectional and lacked control subjects (i.e., without BRAO). Thus, we were unable to confirm a causal relationship between IMT-Bmax, reduced HDL, or female gender and poor VA in BRAO. For the same reason, we were unable to examine the association of these variables with the risk of the development of BRAO. Second, our study lacked patient histories for diabetes, dyslipidemia, and indicators of adiposity (e.g., body mass index and waist-hip ratio). In addition, our study lacked data on the use of medications for hypertension, diabetes, and dyslipidemia. These factors are known to be involved in cardiovascular diseases, and might affect ocular blood flow in BRAO. Third, because BRAO is a relatively rare disease, the number of subjects was small, although it was comparable to previous reports on BRAO [5, 6]. This might have contributed to the low statistical power of some of our findings, such as the lack of a significant difference in IMT-Bmax between the groups. While we included only 14 patients with worse VA outcomes, we included 6 predictors in the multivariate logistic regression model (Table 3). This might have caused overfitting of the model [73]. In addition, the 95% CI for OR for gender (Table 3) was large due to the small sample size, which might have caused statistical bias [74]. Therefore, the results of our multivariate logistic regression model should interpreted with caution. Fourth, we did not carry out echocardiography, and thus cannot confirm that our study did not include patients with emboli originating in the heart, although only a single patient in each VA group had atrial fibrillation. Lastly, our study did not evaluate ocular blood flow, although FA was included as part of diagnosis. Thus, we are unable to confirm the association between the identified risks and ischemic changes in the affected retinas. Further longitudinal studies including larger sample sizes and the measurement of ocular blood flow with optical coherence tomography or laser speckle flowgraphy are required.

In conclusion, we found that increased IMT-Bmax, decreased HDL, and female gender were associated with poor VA at the time of initial presentation in patients with BRAO. This finding promises to provide a useful indicator for making more accurate VA prognoses in patients with BRAO, and enable new insights into the pathomechanisms underlying BRAO.

## Supporting information

S1 Fig Representative images of the IMT-Bmax. IMT-Bmax (A).
(TIF)

S2 Fig Representative images of the IMT-Cmax. IMT-Cmax (A).
(TIF)

S1 Table Comparison of IMT-Bmax and Cmax between affected and unaffected sides of BRAO. (DOCX)

S2 Table Datasheet used in this study. (XLSX)
