# Supplementary figures and images for: Carotid artery intima-media thickness, HDL cholesterol levels, and gender associated with poor visual acuity in patients with branch retinal artery occlusion
Source: PLoS One. 2020 Oct 22;15(10):e0240977. doi: 10.1371/journal.pone.0240977 (PMC7580897; doi:10.1371/journal.pone.0240977)

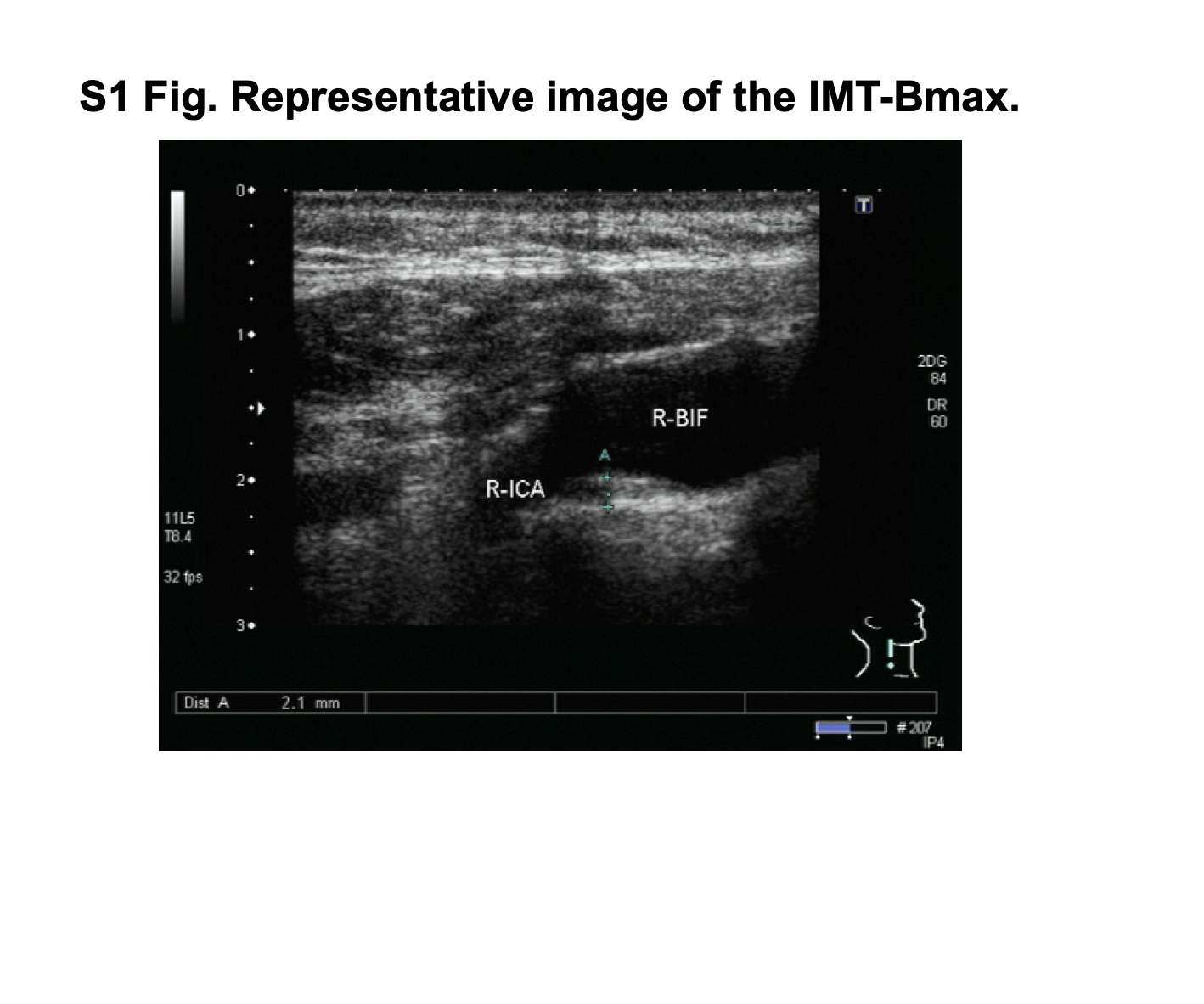

Supplement: S1 Fig — IMT-Bmax (A). (TIF) [file pone.0240977.s001.tif]

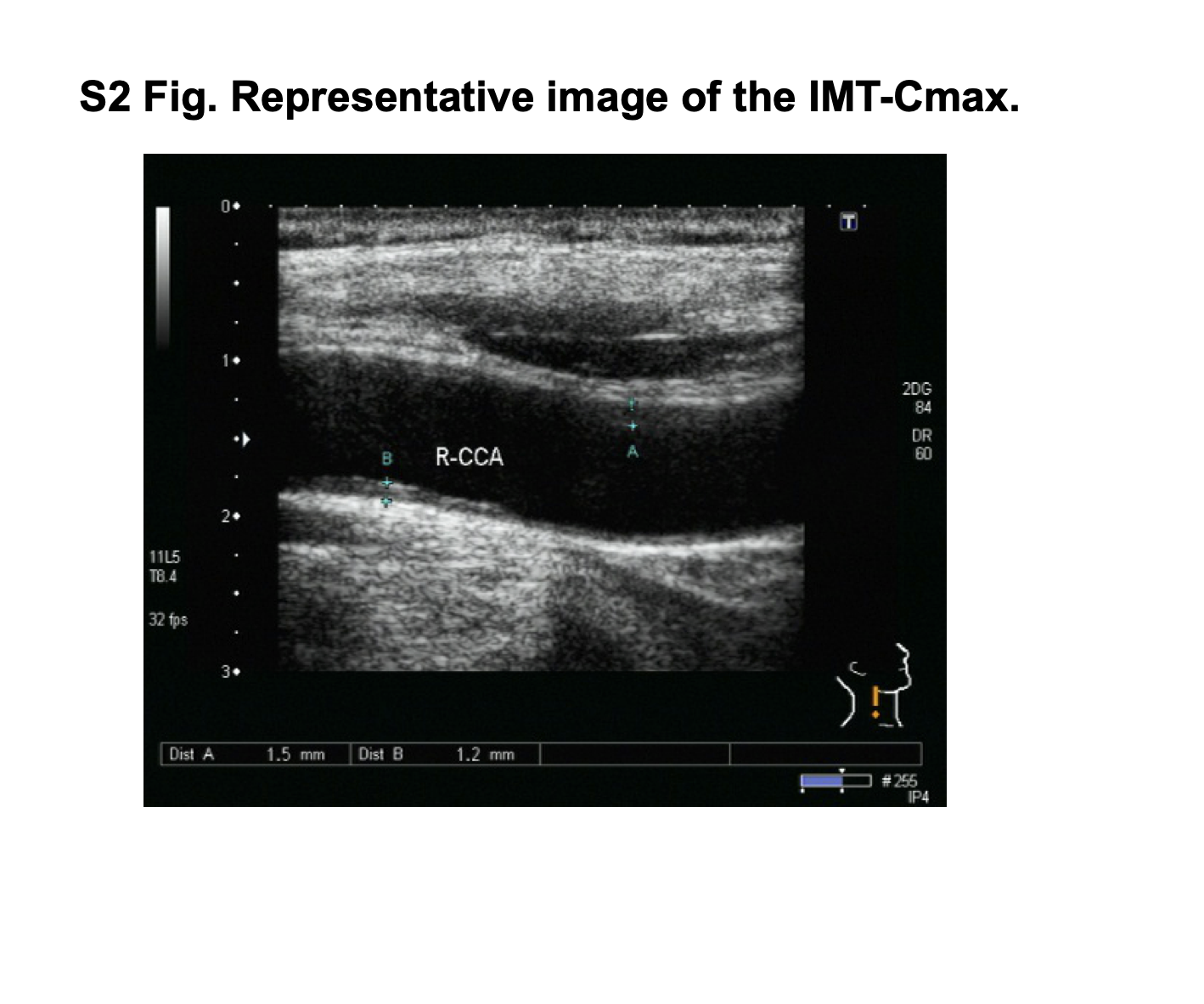

Supplement: S2 Fig — IMT-Cmax (A). (TIF) [file pone.0240977.s002.tif]
