# Supplementary material for: Carotid artery intima-media thickness, HDL cholesterol levels, and gender associated with poor visual acuity in patients with branch retinal artery occlusion
Source: PLoS One. 2020 Oct 22;15(10):e0240977. doi: 10.1371/journal.pone.0240977 (PMC7580897; doi:10.1371/journal.pone.0240977)
Supplement: S1 Table — (DOCX) [file pone.0240977.s003.docx]

**S1 Table. Comparison of IMT-Bmax and Cmax between affected and unaffected sides.**

|  | **Without BRAO** | **With BRAO** | ***P* value** |
| --- | --- | --- | --- |
| **IMT-Bmax (mm)** | 1.70 [1.20, 2.50]^†^ | 1.65 [1.10, 2.53] | 0.707 |
| **IMT-Cmax (mm)** | 1.00 [0.80, 1.50] | 1.20 [0.90, 2.00] | 0.025* |
| IMT-Bmax and IMT-Cmax were compared between BRAO affected and unaffected sides using the Wilcoxon signed-rank test. **P* < 0.05. ^†^One case was excluded from the IMT-Bmax without BRAO due to poor image quality of the carotid artery ultrasound. | | | |
